# Mitigating iftar-related glycemic excursions in adolescents and young adults with type 1 diabetes on MiniMed™ 780G advanced hybrid closed loop system: a randomized clinical trial for adjunctive oral vildagliptin therapy during Ramadan fasting

**DOI:** 10.1186/s13098-023-01232-5

**Published:** 2023-12-07

**Authors:** Nancy Samir Elbarbary, Eman Abdel Rahman Ismail

**Affiliations:** 1 Department of Pediatrics, Faculty of Medicine, Ain Shams University, 25 Ahmed Fuad St. Saint Fatima, Heliopolis, Cairo, 11361 Egypt; 2 Department of Clinical Pathology, Faculty of medicine, Ain shams University, Cairo, Egypt

## Abstract

**Background:**

Ramadan Iftar meal typically causes glucose excursions. Dipeptidyl peptidase-4 inhibitors increase glucagon-like peptide-1 and thus, decrease blood glucose levels with low risk of hypoglycemia.

**Aim:**

To investigate the efficacy and safety of vildagliptin as an add-on therapy on glucose excursions of Iftar Ramadan meals among adolescents and young adults with type 1 diabetes mellitus (T1DM) using advanced hybrid closed-loop (AHCL) treatment.

**Methods:**

Fifty T1DM patients on MiniMed™ 780G AHCL were randomly assigned either to receive vildagliptin (50 mg tablet) with iftar meal during Ramadan month or not. All participants received pre-meal insulin bolus based on insulin-to-carbohydrate ratio (ICR) for each meal constitution.

**Results:**

Vildagliptin offered blunting of post-meal glucose surges (mean difference − 30.3 mg/dL [− 1.7 mmol/L] versus − 2.9 mg/dL [− 0.2 mmol/L] in control group; p < 0.001) together with concomitant exceptional euglycemia with time in range (TIR) significantly increased at end of Ramadan in intervention group from 77.8 ± 9.6% to 84.7 ± 8.3% (p = 0.016) and time above range (180–250 mg/dL) decreased from 13.6 ± 5.1% to 9.7 ± 3.6% (p = 0.003) without increasing hypoglycemia. A significant reduction was observed in automated daily correction boluses and total bolus dose by 23.9% and 16.3% (p = 0.015 and p < 0.023, respectively) with less aggressive ICR settings within intervention group at end of Ramadan. Coefficient of variation was improved from 37.0 ± 9.4% to 31.8 ± 7.1%; p = 0.035). No severe hypoglycemia or diabetic ketoacidosis were reported.

**Conclusion:**

Adjunctive vildagliptin treatment mitigated postprandial hyperglycemia compared with pre-meal bolus alone. Vildagliptin significantly increased TIR while reducing glycemic variability without compromising safety.

*Trial registration* This trial was registered under ClinicalTrials.gov Identifier no. NCT06021119.

## Highlights

- This is the first clinical trial assessing oral vildagliptin in T1DM patients using AHCL system during Ramadan fasting.
- Vildagliptin mitigated postprandial hyperglycemia, glycemic variability and time in ranges.
- Vildagliptin concomitantly reduced prandial insulin delivery and total daily insulin dose during AHCL treatment.
- Vildagliptin added to pre-meal insulin bolus in MiniMed™ 780G system allows patients to reach all the international targets for T1DM during fasting without compromising safety.
- The use of adjunctive vidagliptin therapy may ease the burden placed on AHCL systems to mitigate postprandial glycemic excursions.

## Introduction

Current management of people with type 1 diabetes mellitus (T1DM) on intensive insulin therapy recognizes carbohydrates as the most important determinant of postprandial glycaemia; hence, worldwide guidelines recommend carbohydrates counting for determining pre-prandial insulin doses [1, 2]. Currently, the insulin to carbohydrate ratio (ICR) is frequently used to calculate the meal insulin dose. However, ICRs are considered difficult, ineffective and inaccurate for some patient, with an estimation error of around 20% in adults [3, 4] demonstrating only modest improvements in glycated hemoglobin (HbA1c) [5]. Furthermore, a meta-analysis study of ICR use in children and adolescence showed no statistical improvements in outcomes [6]. This lack of effectiveness and the wide variability using ICRs suggests it should be improved upon [7].

In addition, research has identified a significant contribution of other dietary factors, including fat and protein to this postprandial glycemic variability [8, 9]. It has been demonstrated that fat and/or protein when consumed in combination with carbohydrate increase postprandial glycemia and delay gastric emptying leading to a lag in glucose absorption [10]. In the absence of appropriate insulin adjustment, this manifests clinically as late sustained postprandial hyperglycemia [8, 11].

The development of continuous glucose monitoring (CGM) has led to the introduction of automated insulin delivery systems, known as closed-loop insulin delivery systems. These systems use a mathematical dosing algorithm that takes real-time data from a continuous glucose monitor to titrate infusion by an insulin pump [12]. Closed-loop systems improve glycemic control compared with pump therapy and sensor-augmented pump therapy [12, 13]; however, users still have to manually count and enter the carbohydrate content of meals to determine prandial insulin boluses. These systems are described as advanced hybrid closed-loop systems (AHCL) rather than fully closed-loop systems because of the manual entry of pre-meal boluses [14]. The Minimed™ 780G AHCL system adapts basal infusion rates and delivers auto-correction boluses in order to achieve a user-decided glucose target [15]. The increasing, widespread use of this technology has been accompanied by an unprecedented level of interest in the dynamics of the postprandial glycemic profile and in turn, a demand for clinical explanations for aberrant postprandial glycemic patterns [16].

Ramadan fasting is a pillar of the Islamic faith observed by Muslims all around the world. Nutritionally, it involves abstaining from food and water from dawn to sunset and is therefore, associated with many physiological effects that can negatively impact diabetes control [17]. When fasting during Ramadan, there is a dramatic change in dietary patterns in comparison to the other months of the year. Health issues can arise due to improper eating habits and reduced physical activity [18, 19].

The nutritional composition of the Egyptian Iftar meal is characterized by both high glycemic index carbohydrate and high fat components [20]. Unhealthy nutrition habits that commonly develop include the consumption of unusually large meals at Iftar (frequently containing more than 1500 calories with significant amounts of highly processed carbohydrates and fried foods with trans-fat margarine or oils rich in saturated fat) result in severe postprandial hyperglycemia [21]. In addition, eating desserts loaded with sugar after Iftar as dates, apricot juice can lead to a prolonged period of postprandial hyperglycemia [22].

Technological advancements and oral adjuncts to insulin therapies are starting to be licensed for the use of people with T1DM. This leads to the question of whether tight glucose control is becoming solely a matter of technique or whether a combination of technique and novel adjunct therapies in addition to insulin might achieve the best effect on glucose variability for people with T1DM [23].

Dipeptidyl peptidase-4 (DPP-4) inhibitors increase the serum concentrations of glucagon-like peptide-1 (GLP-1), which promotes glucose-response insulin secretion and inhibits glucagon secretion from alpha cells [24]. DDP-4 inhibitors have been suggested as an adjunctive treatment because of their mechanisms of action. Being a member of the islet enhancer class, vildagliptin is a potent and selective DPP-4 inhibitor that leads to increased fasting and postprandial endogenous levels of the incretin hormones GLP-1 and glucose-dependent insulinotropic polypeptide (GIP) [25]. Consequently, vildagliptin enhances the sensitivity of beta cells to glucose and thus, improved glucose-dependent insulin secretion [26]. Treatment with vildagliptin 50–100 mg daily in patients with type 2 diabetes mellitus (T2DM) significantly improved markers of beta cell function including homeostasis model assessment-ß (HOMA-3), proinsulin to insulin ratio and measures of beta cell responsiveness from the frequently-sampled meal tolerance test [27–29].

By increasing endogenous GLP-1 levels, vildagliptin also enhances the sensitivity of alpha cells to glucose and results in more glucose-appropriate glucagon secretion. The enhanced increase in the insulin/glucagon ratio during hyperglycemia leads to decreased fasting and postprandial hepatic glucose production and reduced glycemia [30]. The known effect of increased GLP-1 levels delaying gastric emptying is not observed with vildagliptin treatment. It has been reported that vildagliptin improved glycemic control in T2DM when given as monotherapy or when used in combination with metformin, a sulphonylurea, and a thiazolidinedione, as measured by clinically relevant reductions in HbA1c from baseline at study endpoint [31].

There have been only a few randomized controlled studies that investigated the efficacy and safety of DPP-4 inhibitors as an add-on drug in patients treated with basal insulin. An earlier study showed that vildagliptin treatment markedly decreased the post-meal glucagon excursion in insulinopenic patients with T1DM. Furthermore, the magnitude of the effect of vildagliptin to suppress post-meal glucagon secretion in patients with T1DM is similar to that observed in patients with T2DM [32]. In a 4 weeks double-blind, randomized, placebo-controlled trial, vildagliptin improved glycemia and inhibited glucagon levels during meal ingestion but sustained glucagon counterregulation during hypoglycemia indicating its beneficial use in T1DM as an add-on to insulin therapy without increasing the risk for hypoglycemia [33]. Recently, it has been shown that vildagliptin in combination with rapamycin significantly enhanced the insulin mimetic effect of rapamycin in patients with T1DM possibly by improving postprandial glucagon secretion and insulin sensitivity. Vildagliptin also induced some specific hormonal and immunological modification that blunted the effect of rapamycin on GLP-1, ghrelin and adipsin levels and was well tolerated [34].

However, up till now, no randomized controlled studies have investigated the use of DPP-4 inhibitors as an add-on drug in patients treated with AHCL system or during Ramadan fasting. Therefore, we conducted a one-month randomized control trial to investigate the efficacy and safety of vildagliptin as an add-on therapy among adolescents and young adults with T1DM on glucose excursions of Iftar Ramadan meals and glucometrics during AHCL treatment.

## Materials and methods

This prospective, open label, single center, randomized-controlled intervention non-inferiority trial compared insulin aspart bolus plus vildagliptin and insulin aspart bolus alone using AHCL. Established T1DM patients who met the inclusion criteria were recruited from the regular attendants of Diabetes Clinic. Those who observed fasting in Ramadan 2023 using AHCL systems were invited to participate in a real-world setting with no impact on routine clinical care. The study was approved from the local ethical committee and all participants or their legal representatives provided signed, informed consent after being informed about the study before any trial-related activities. Reporting of the study conforms to Consolidated Standards of Reporting Trials 2010 statement [35].

Inclusion criteria were patients with T1DM [36] for at least 1 year, aged 12–27 years and using MiniMed™ 780G AHCL system (Medtronic, Northridge, CA, USA) with Guardian™ 3 sensor or Guardian™ 4 calibration-free sensor MiniMed and Guardian link transmitter initiated at least 6 months before the study, patients with minimum daily insulin requirement of more than 8 units, willingness and ability to adhere to the study protocol, access to the internet and a computer system that met requirements for uploading the study pump data. Insulin Aspart (NovoRapid®, Novo Nordisk, Copenhagen, Denmark) was used in all patients on MiniMed™ 780G AHCL system.

Exclusion criteria were patients with any microvascular or macrovascular complications, pregnancy, lactation and those who had a point-of-care screening HbA1c > 10.0% (86 mmol/mol), hypoglycemic unawareness or recurrent severe hypoglycemic episode in the last 6 months prior to recruitment as well as recurrent diabetic ketoacidosis (DKA, more than 2 episodes in the previous 6 months). Patients with any chronic medical condition, current use of medications (other than insulin) that are known to affect blood glucose level or those who had prior adverse reactions to the adjunctive agent under study were also excluded.

### Sample size

Sample size was calculated using PASS program version 15, setting alpha error at 5% and power at 90%. After reviewing literature, no similar research has been done before. Therefore, assuming the mean percent change in postprandial blood glucose at 2 h among vildagliptin group was − 8.3% compared with − 2.8% in the control group; based on this, the needed sample was 15 cases per group. We included 25 patients in each group (with a total of 50 patients) to increase the power of study and take into consideration the drop-out rate.

### Ramadan AHCL protocol steps

#### I. Pre-Ramadan assessment

##### Safe fasting instructions for patients and health care givers

A pre-Ramadan assessment took place 2 to 3 weeks before the start of Ramadan. The studied patients with T1DM were subjected to detailed medical history taking, risk assessment score, and thorough clinical examination focusing on age of onset of diabetes, duration on insulin pump therapy and dietary intake. Anthropometric measurements were recorded.

In addition, nutrition plan, timing of breaking the fast, standard self-management of diabetes related emergency, ketone measurement, hypoglycemia and hyperglycemia treatment guidelines were all discussed. All patients were instructed to end their fast immediately if blood glucose reaches < 70 mg/dL (< 3.9 mmol/L), symptomatic hypoglycemia or if they feel unwell in any hours after the start of the fast. The fast should also be broken if blood glucose exceeds 300 mg/dL (> 16.6 mmol/L) [22] and patients were advised to check the pump and the infusion site with administration of correction dose.

##### AHCL procedures instructions

- Step 1: Competency assessment: explaining individuals’ responsibilities and commitments (attending all training session, meal bolus timing, calibrating the system, responding to alerts and alarms, set/reservoir change, CareLink mobile application, Auto Mode usage) were discussed with patients and healthcare givers. AHCL system was used continuously for 4 weeks and in case of AHCL exit, the participants were instructed to perform the actions recommended by the pump to re-enter the system.
- Step 2: Downloads were reviewed for the settings before Ramadan including assessment and progress report, weekly/daily review report, device settings report, meal bolus wizard and adherence report. System adjustments were performed whenever necessary to meet the currently agreed-upon targets for CGM-derived metrics.

#### II. Randomization of the study population and AHCL setting adjustments during Ramadan

**Fig. 1:**
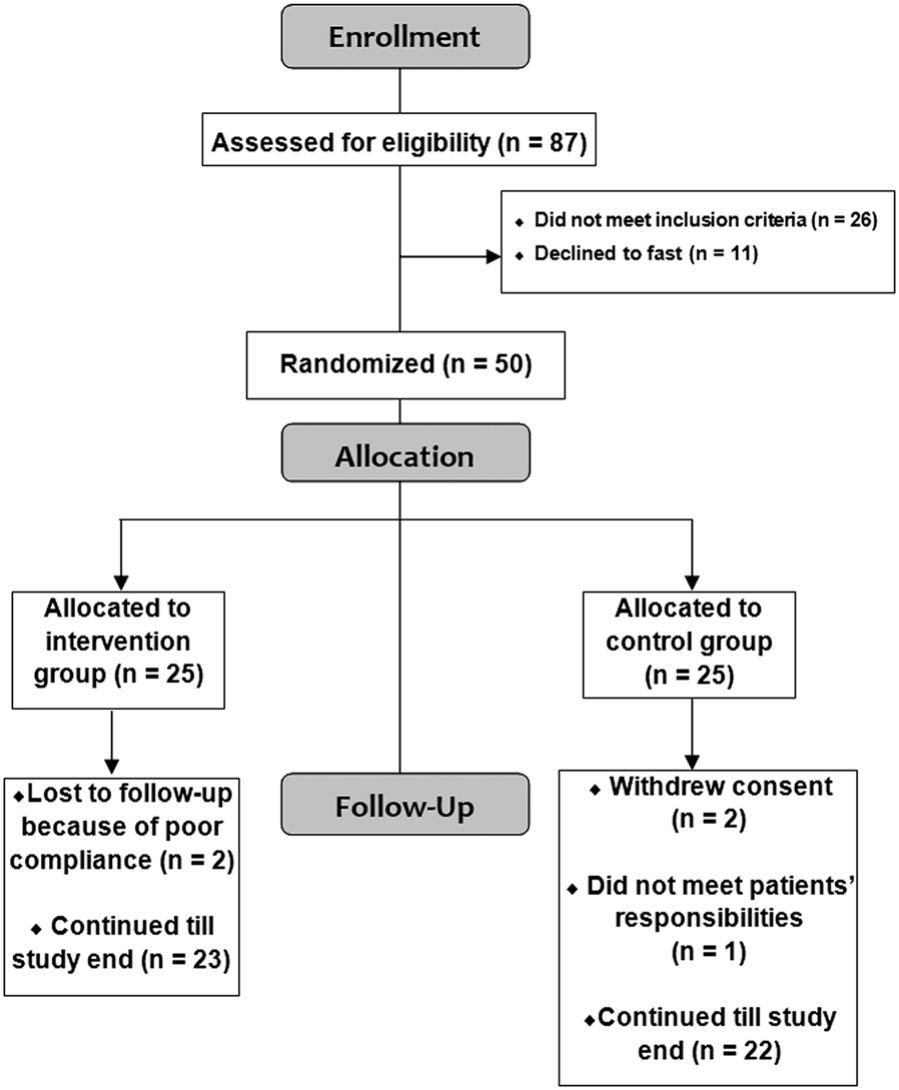
CONSORT flow diagram for recruitment of patients with T1DM on AHCL system. Fifty patients were randomly allocated to either vildagliptin or control groups during Ramadan fasting

A total of 87 T1DM patients were evaluated two months preceding Ramadan. Twenty-six patients did not meet inclusion criteria and 11 patients elected not to fast while the remaining eligible 50 patients who chose to fast were randomized into either intervention group (n = 25) and control group (n = 25). A simple randomization method was used. Five patients dropped out (two in the intervention group lost to follow-up because of poor compliance while two in the control group withdrew consent and one did not meet patients’ responsibilities) (Fig. 1).

Patients in the intervention group received vildagliptin (Gliptus 50 mg tablet, manufactured by Horus for pharmaceutical industries, affiliate of EVA Group limited, Egypt) with iftar meal for the whole month of Ramadan (4 weeks) in addition to pre-meal insulin iftar bolus. The active substance of the tablet is 50 mg vildagliptin. The other ingredients are lactose anhydrous, microcrystalline cellulose, sodium starch glycollate and magnesium stearate. The control group only administered pre-meal insulin iftar bolus without add on drug therapy.

AHCL system was adjusted for both groups as follows: algorithm glucose were kept the same as before Ramadan with glucose target 100 mg/dL (5.5 mmol/L), active insulin time (AIT) was set for 2 h and the pre-iftar insulin bolus was determined in both groups based on each participant’s ICR for that given meal. However, ICR settings in Ramadan were adjusted according to blood glucose readings during follow-up in each group thereafter.

Both groups were instructed to bolus insulin before meal and better avoid vigorous physical activity particularly during the few hours before the sunset meal. Nevertheless, while in Auto Mode, participants may set a temporary sensor glucose target (Temp Target) of 150 mg/dL (8.3 mmol/L) for situations in which they would like their target to be temporarily higher. Clinical and technical support was always available with text messaging and phone calls during the study.

The patient uploaded the AHCL system on Carelink Personal Software on days 1, 7, 14, 21, 30 and whenever indicated during Ramadan. Glucose and insulin metrics were analyzed 2 weeks before, weekly during Ramadan and at the end of Ramadan period. Percentages of time in range (TIR) 70–180 mg/dL (3.9–10 mmol/L), time below range (TBR) 70 mg/dL (3.9 mmol/L) and 54 mg/dL (3.0 mmol/L), and time above range (TAR) 180 mg/dL (10.0 mmol/L) and 250 mg/dL (13.9 mmol/L) were calculated. CGM-captured hypoglycemia was considered as one episode when glucose fell to < 70 mg/dL (3.9 mmol/L) for at least 15 consecutive minutes. Glycemic control was estimated as glucose management indicator (GMI) using a minimum of 14 days of CGM data. Glucose variability was estimated by the calculation of the coefficient of variation (CoV) of CGM readings.

### Follow-up and endpoints

All patients were clinically followed-up every week during Ramadan for evaluating compliance to study treatment and monitoring signs of any potential adverse effects. The primary outcome of the study was the peak postprandial plasma glucose (PPG) level calculated by pooling data for up to 3 h after the start of iftar meal. Secondary outcomes included TIR change from baseline to end of Ramadan, changes in TBR, TAR, average sensor glucose (SG) readings, CoV, number of full fasted days that were completed during the month and fast-breaking events. Further endpoints included the degree of insulin dose reduction during automated insulin delivery after vildagliptin. Safety outcomes were measured by recording episodes of severe hypoglycemia and/or DKA both requiring medical attention or emergency hospital visits for diabetes-related problems before and during Ramadan. Gastrointestinal symptoms were assessed for the intervention group. Additionally, any adverse events that occurred during the study were recorded.

### Statistical analysis

Data were collected, revised, coded and entered to the Statistical Package for Social Science (SPSS) software version 27 (IBM SPSS Statistics, IBM Corporation, Chicago, IL, USA). This study was exploratory, and no power calculations were required. The statistical analysis plan followed the completion of the last patient’s last visit but before the final dataset was reviewed and analyzed. Data analysis was performed for the entire study population. Insulin and CGM data were collected from CareLink Therapy Management Software during the study. Kolmogrov-Smirnov test was used to examine the normal distribution of variables. To detect baseline differences between the intervention and control groups as regards age, sex, body mass index (BMI) and disease duration, we used independent sample Student’s *t* test for quantitative parametric data and Mann–Whitney test for non-parametric data while Chi-Square (X^2^) test was used for qualitative variables. To identify within-group changes (before and after Ramadan), we applied paired-samples t tests for quantitative parametric data. Analysis of covariance (ANCOVA) was performed to compare mean values between groups adjusted for differences in baseline measures. Mean differences (95% confidence interval [CI]) between groups were compared using Mann–Whitney test.

A p value < 0.05 was considered significant in all analyses.

## Results

### Characteristics of the study population

A total of 50 T1DM patients on MiniMed™ 780G completed the study; 24 males and 26 females. The mean age of vildagliptin group was 16.5 ± 3.5 years while that of the control group was 17.1 ± 4.2 years (Table 1). The mean diabetes duration was 7.3 ± 2.6 years and mean time on insulin pump therapy (AHCL system) was 1.87 ± 0.7 years in the study population. Baseline mean insulin dose (IU/kg/day) of vildagliptin group was 1.27 ± 0.28 and in control group was 1.34 ± 0.31; p = 0.395).

**Table 1:** Baseline clinical data of the randomized population with type 1 diabetes on MiniMed™ 780G insulin pump

Variable	Vildagliptin group (n = 25)	Control group (n = 25)	p value
Age (years)			
Mean ± SD	16.5 ± 3.5	17.1 ± 4.2	0.586
Gender, n (%)			
Male	13 (52)	11 (44)	0.571
Female	12 (48)	14 (56)	
Weight SDS			
Median (IQR)	0.25 (0.08–1.43)	0.31 (0.18–1.23)	0.892
BMI SDS			
Median (IQR)	1.21 (0.10–1.76)	1.17 (0.4–1.6)	0.899
Diabetes duration (years)			
Mean ± SD	7.52 ± 2.65	7.12 ± 2.45	0.582
AHCL therapy duration (years)			
Mean ± SD	1.93 ± 0.86	1.72 ± 0.75	0.362

*BMI* body mass index, *SDS* standard deviation score, *AHCL* Advanced Hybrid Closed Loop System

All participants had excellent glycemic levels before Ramadan as shown by GMI (estimated A1C [eA1C]) level and optimal TIR. The number of days fasted was 27.8 ± 0.5 days in the intervention group and 28.0 ± 0.6 days in the control group (p = 0.217) with an average fasting of around 12–13 h/day. AHCL system performance at baseline is shown in Table 2. No significant baseline differences were found between the intervention and control group (p > 0.05) (Tables 1 and 2).

**Table 2:** Baseline MiniMed™ 780G system data among the enrolled patients with T1DM

Variable	Vildagliptin group (n = 25)	Control group (n = 25)	p value
Average SG (mg/dL)	151.4 ± 20.5	157.9 ± 22.3	0.289
Average SG (mmol/L)	8.4 ± 1.1	8.8 ± 1.2	0.228
GMI (eA1C %)	6.94 ± 0.63	6.88 ± 0.55	0.721
GMI (eA1C mmol/moL)	52.2 ± 7.3	51.6 ± 6.8	0.757
CoV (%)	37.0 ± 9.4	37.8 ± 9.1	0.761
TIR 70–180 mg/dL (3.9–10 mmol/L) (%)	77.8 ± 9.6	78.9 ± 9.1	0.679
TBR < 70 mg/dL (< 3.9 mmol/L) (%)	3.2 ± 0.8	3.5 ± 1.0	0.247
TBR < 54 mg/dL (< 3.0 mmol/L) (%)	0.3 ± 0.14	0.3 ± 0.12	1.000
TAR 180–250 mg/dL (10.0–13.9 mmol/L) (%)	13.6 ± 5.1	13.1 ± 4.2	0.707
TAR > 250 mg/dL (> 13.9 mmol/L) (%)	5.1 ± 1.3	4.5 ± 1.0	0.072
Total daily dose (U/day)	49.8 ± 10.2	48.3 ± 8.9	0.582
Bolus amount (U/day)	27.6 ± 6.7	26.7 ± 7.3	0.652
Auto correction amount (day)	6.7 ± 1.5	6.3 ± 1.4	0.335
Auto Basal/Basal amount (day)	22.2 ± 7.8	21.6 ± 6.8	0.773
BG at the start of the meal (mg/dL)	117.1 ± 36.3	120.8 ± 29.2	0.693
BG at the start of the meal (mmol/L)	6.5 ± 2.0	6.7 ± 1.6	0.689
BG at 60 min from the start of the meal (mg/dL)	178.2 ± 37.4	182.6 ± 35.1	0.669
BG at 60 min from the start of the meal (mmol/L)	9.9 ± 2.1	10.1 ± 1.9	0.719
BG at 120 min from the start of the meal (mg/dL)	207.5 ± 51.3	219.3 ± 36.2	0.352
BG at 120 min from the start of the meal (mmol/L)	11.5 ± 2.8	12.2 ± 2.0	0.325
BG at 180 min from the start of the meal (mg/dL)	183.7 ± 46.1	187.8 ± 35.8	0.727
BG at 180 min from the start of the meal (mmol/L)	10.2 ± 2.6	10.4 ± 1.9	0.752
Carbohydrates (g/day)	156.2 ± 28.7	161.2 ± 25.3	0.517
ICR (g)	10.2 ± 2.6	10.8 ± 3.0	0.454
Smart gaurd/week auto mode (%)	95.5 ± 4.1	96.1 ± 4.4	0.621
Sensor wear (%)	96.8 ± 3.6	95.7 ± 3.9	0.305
Exit from AHCL per patient (n/week)	1.3 ± 0.6	1.2 ± 0.5	0.525
BG calibration (n/day)	3.5 ± 0.6	3.4 ± 0.6	0.559
Set change (n of days)	3.3 ± 1.1	3.4 ± 1.2	0.761
Reservoir change (n of days)	3.4 ± 0.7	3.1 ± 0.5	0.088

*T1DM* type 1 diabetes mellitus, *SG* sensor glucose, *GMI* glucose management indicator, *eA1C* estimated A1C, *CoV* coefficient of variation, *BG* blood glucose, *TIR* time in range, *TBR* time below range, *TAR* time above range, *ICR* insulin to carb ratio

### Effect of adjunctive vildagliptin therapy on postprandial glucose excursions and glycemic control delivered by AHCL system during Ramadan

The effect of treatment in blunting and delaying meal-stimulated increments in plasma glucose levels was observed. Glucose values closer to the start of the meal (prior to the meal) were similar in both intervention and control groups. At 60 min from the start of the meal, the mean glucose values were significantly lower in the intervention group (vildagliptin + bolus insulin) compared with baseline levels and with the control group. The mean peak postprandial glucose level at 120 min after Iftar meal in the intervention group was 177.2 ± 45.7 mg/dL (9.8 ± 2.5 mmol/L) compared with pre-Ramadan levels 207.5 ± 51.3 mg/dL (11.5 ± 2.8 mmol/L) (p = 0.033) and also compared with 216.4 ± 33.7 mg/dL (12.0 ± 1.8 mmol/L) in the control group (p = 0.002). The mean difference (95% CI) of postprandial glucose level at 120 min was − 30.3 (− 57.928 to − 2.672) mg/dL among vildagliptin group versus − 2.9 (− 22.789–16.989) mg/dL in the control group. Additionally, at 180 min from the start of the meal, the glucose values were 149.3 ± 38.4 mg/dL (8.3 ± 2.1 mmol/L) and 176.7 ± 39.1 mg/dL (9.8 ± 2.2 mmol/L) in intervention and control group, respectively (p = 0.002) (Tables 3 and 4). These values suggest a beneficial effect of vildagliptin immediately following the meal bolus, which diminished over time by three hours.

**Table 3:** Comparison between vildagliptin and control groups as regards MiniMed™ 780G glucometrics and glycemic excursions before and at the end of Ramadan among the enrolled patients with T1DM

Variable	Vildagliptin group		p value^a^	Control group		p value^a^	p value^b^
	Before Ramadan (n = 25)	End of Ramadan (n = 23)		Before Ramadan (n = 25)	End of Ramadan (n = 22)		
Average SG (mg/dL)	151.4 ± 20.5	140.1 ± 12.5	0.025	157.9 ± 22.3	168.2 ± 19.6	0.113	< 0.001
Average SG (mmol/L)	8.4 ± 1.1	7.8 ± 0.7	0.032	8.8 ± 1.2	9.3 ± 1.1	0.139	< 0.001
GMI (eA1C %)	6.94 ± 0.63	6.42 ± 0.58	0.005	6.88 ± 0.55	6.97 ± 1.1	0.695	0.045
GMI (eA1C mmol/moL)	52.2 ± 7.3	47.1 ± 5.9	0.015	51.6 ± 6.8	52.8 ± 6.1	0.481	0.002
CoV (%)	37.0 ± 9.4	31.8 ± 7.1	0.035	37.8 ± 9.1	41.6 ± 9.8	0.194	< 0.001
TIR 70–180 mg/dL (3.9–10 mmol/L) (%)	77.8 ± 9.6	84.7 ± 8.3	0.016	78.9 ± 9.1	79.1 ± 8.5	0.687	0.036
TBR < 70 mg/dL (< 3.9 mmol/L) (%)	3.2 ± 0.8	2.9 ± 0.9	0.212	3.5 ± 1.0	3.1 ± 0.8	0.112	0.428
TBR < 54 mg/dL (< 3.0 mmol/L) (%)	0.3 ± 0.14	0.3 ± 0.13	0.875	0.3 ± 0.12	0.3 ± 0.11	0.971	0.986
TAR 180–250 mg/dL (10.0–13.9 mmol/L) (%)	13.6 ± 5.1	9.7 ± 3.6	0.003	13.1 ± 4.2	13.2 ± 4.4	0.829	0.005
TAR > 250 mg/dL (> 13.9 mmol/L) (%)	5.1 ± 1.3	2.4 ± 0.9	< 0.001	4.5 ± 1.0	4.3 ± 1.1	0.479	< 0.001
Total daily dose (U/day)	49.8 ± 10.2	43.4 ± 8.4	0.023	48.3 ± 8.9	52.4 ± 9.9	0.164	0.002
Bolus amount (U/day)	27.6 ± 6.7	23.1 ± 5.8	0.015	26.7 ± 7.3	32.6 ± 8.7	0.187	< 0.001
Auto correction amount (day)	6.7 ± 1.5	5.1 ± 1.2	< 0.001	6.3 ± 1.4	7.1 ± 1.6	0.084	< 0.001
Auto Basal/Basal amount (day)	22.2 ± 7.8	20.3 ± 4.8	0.292	21.6 ± 6.8	19.8 ± 7.7	0.397	0.689
BG at the start of the meal (mg/dL)	117.1 ± 36.3	112.0 ± 30.7	0.213	120.8 ± 29.2	114.5 ± 32.1	0.257	0.584
BG at the start of the meal (mmol/L)	6.5 ± 2.0	6.2 ± 1.7	0.512	6.7 ± 1.6	6.4 ± 1.8	0.548	0.697
BG at 60 min from the start of the meal (mg/dL)	178.2 ± 37.4	159.1 ± 26.3	0.041	182.6 ± 35.1	188.3 ± 36.3	0.518	0.005
BG at 60 min from the start of the meal (mmol/L)	9.9 ± 2.1	8.8 ± 1.4	0.036	10.1 ± 1.9	10.4 ± 2.0	0.624	0.004
BG at 120 min from the start of the meal (mg/dL)	207.5 ± 51.3	177.2 ± 45.7	0.033	219.3 ± 36.2	216.4 ± 33.7	0.715	0.002
BG at 120 min from the start of the meal (mmol/L)	11.5 ± 2.8	9.8 ± 2.5	0.032	12.2 ± 2.0	12.0 ± 1.8	0.728	0.003
BG at 180 min from the start of the meal (mg/dL)	183.7 ± 46.1	149.3 ± 38.4	0.005	187.8 ± 35.8	176.7 ± 39.1	0.357	0.021
BG at 180 min from the start of the meal (mmol/L)	10.2 ± 2.6	8.3 ± 2.1	0.006	10.4 ± 1.9	9.8 ± 2.2	0.341	0.025
Carbohydrates (g/day)	156.2 ± 28.7	211.6 ± 35.3	< 0.001	161.2 ± 25.3	226.5 ± 39.2	< 0.001	0.214
ICR (g)	10.2 ± 2.6	11.9 ± 3.4	0.063	10.8 ± 3.0	7.1 ± 2.9	< 0.001	< 0.001
Smart gaurd/week auto mode (%)	95.5 ± 4.1	97.4 ± 5.7	0.194	96.1 ± 4.4	97.7 ± 4.8	0.167	0.412
Sensor wear (%)	96.8 ± 3.6	97.1 ± 4.5	0.815	95.7 ± 3.9	97.8 ± 4.1	0.085	0.625
Exit from AHCL per patient (n/week)	1.3 ± 0.6	1.1 ± 0.4	0.186	1.2 ± 0.5	1.4 ± 0.7	0.174	0.094
BG calibration (n/day)	3.5 ± 0.6	3.8 ± 0.7	0.221	3.4 ± 0.6	3.7 ± 0.6	0.125	0.616
Set change (n of days)	3.3 ± 1.1	3.6 ± 1.0	0.307	3.4 ± 1.2	3.5 ± 1.1	0.726	0.759
Reservoir change (n of days)	3.4 ± 0.7	3.5 ± 0.5	0.568	3.1 ± 0.5	3.3 ± 0.6	0.218	0.261
Number of days fasting completed	–	27.8 ± 0.5	–	–	28.0 ± 0.6	–	0.217

*T1DM* type 1 diabetes mellitus, *SG* sensor glucose, *GMI* glucose management indicator, *eA1C* estimated A1C, *CoV* coefficient of variation, *BG* blood glucose, *TIR* time in range, *TBR* time below range, *TAR* time above range, *ICR* insulin to carb ratio

^a^P value was obtained from paired-samples t test

^b^P value was obtained using Analysis of covariance (ANCOVA)

**Table 4:** Treatment effect between vildagliptin and control groups in patients with T1DM on AHCL illustrating mean difference (95% confidence interval) of glucometrics and glycemic excursions before and at the end of Ramadan

Variable	Mean difference (95% CI)		p value
	Vildagliptin group (n = 23)	Control group (n = 22)	
Average SG (mg/dL)	− 12.5 (− 20.955 to − 1.645)	10.3 (− 1.639 to 22.239)	< 0.001
Average SG (mmol/L)	− 0.6 (− 1.124 to − 0.075)	0.5 (− 0.155 to 1.155)	< 0.001
GMI (eA1C %)	− 0.52 (− 0.864 to 0.176)	0.09 (− 0.405 to 0.585)	< 0.001
GMI (eA1C mmol/moL)	− 5.1 (− 8.874 to − 1.326)	1.2 (− 2.473 to 4.873)	< 0.001
CoV (%)	− 5.2 (− 9.937 to − 0.463)	3.8 (− 1.578 to 9.178)	< 0.001
TIR 70–180 mg/dL (3.9–10 mmol/L) (%)	6.9 (1.797 to 12.003)	0.2 (− 4.807 to 5.207)	< 0.001
TBR < 70 mg/dL (< 3.9 mmol/L) (%)	− 0.3 (− 0.784 to 0.184)	− 0.4 (− 0.915 to 0.115)	0.161
TBR < 54 mg/dL (< 3.0 mmol/L) (%)	0.0 (− 0.076 to 0.076)	0.0 (− 0.066 to 0.066)	1.000
TAR 180–250 mg/dL (10.0–13.9 mmol/L) (%)	− 3.9 (− 6.41 to − 1.39)	0.1 (− 2.346 to 2.546)	< 0.001
TAR > 250 mg/dL (> 13.9 mmol/L) (%)	− 2.7 (− 3.336 to − 2.064)	0.10 (− 0.472 to 0.672)	< 0.001
Total daily dose (U/day)	− 6.4 (− 11.714 to − 1.086)	4.1 (− 1.253 to 9.453)	< 0.001
Bolus amount (U/day)	− 4.5 (− 8.064 to − 0.936)	5.9 (1.333 to 10.467)	< 0.001
Auto correction amount (day)	− 1.6 (− 3.372 to − 0.828)	0.8 (− 0.054 to 1.65)	< 0.001
Auto Basal/Basal amount (day)	− 1.9 (− 5.583 to 1.783)	− 1.8 (− 5.931 to 2.331)	0.857
BG at the start of the meal (mg/dL)	− 5.1 (− 24.218 to 14.018)	− 6.3 (− 23.75 to 11.15)	0.643
BG at the start of the meal (mmol/L)	− 0.3 (− 1.356 to 0.756)	− 0.3 (− 1.268 to 0.668)	1.000
BG at 60 min from the start of the meal (mg/dL)	− 19.1 (− 37.486 to − 0.714)	5.7 (− 14.605 to 26.005)	< 0.001
BG at 60 min from the start of the meal (mmol/L)	− 1.1 (− 2.115 to − 0.085)	0.3 (− 0.809 to 1.409)	< 0.001
BG at 120 min from the start of the meal (mg/dL)	− 30.3 (− 57.928 to − 2.672)	− 2.9 (− 22.789 to 16.989)	< 0.001
BG at 120 min from the start of the meal (mmol/L)	− 1.7 (− 3.209 to − 0.191)	− 0.2 (− 1.282 to 0.882)	< 0.001
BG at 180 min from the start of the meal (mg/dL)	− 34.4 (− 58.527 to − 10.27)	− 11.1 (− 32.418 to 0.218)	< 0.001
BG at 180 min from the start of the meal (mmol/L)	− 1.9 (− 3.244 to − 0.556)	− 0.6 (− 1.769 to 0.569)	< 0.001
Carbohydrates (g/day)	55.4 (37.11 to 73.69)	62.3 (46.53 to 79.06)	0.194
ICR (g)	1.7 (− 0.021 to 3.421)	− 3.7 (− 5.378 to − 2.022)	< 0.001

*T1DM* type 1 diabetes mellitus, *AHCL* Advanced Hybrid Closed Loop System, *SG* sensor glucose, *GMI* glucose management indicator, *eA1C* estimated A1C, *CoV* coefficient of variation, *BG* blood glucose, *TIR* time in range, *TBR* time below range, *TAR* time above range, *ICR* insulin to carb ratio

The average SG was significantly lower in the intervention group compared with the control group; 140.1 ± 12.5 mg/dL (7.8 ± 0.7 mmol/L) versus 168.2 ± 19.6 mg/dL (9.3 ± 1.1 mmol/L); p < 0.001. This mean SG during AHCL equates to a GMI of 6.42 ± 0.58% (47.1 ± 5.9 mmol/moL) versus 6.97 ± 1.1% (52.8 ± 6.1 mmol/moL); p = 0.045 with a mean GMI difference (95% CI) − 5.1 (− 8.874 to − 1.326) mmol/moL compared with 1.2 (− 2.473–4.873) mmol/moL; p < 0.001. The glycemic variability was lower when patients received vildagliptin where CoV differed when comparing the end versus start of Ramadan (31.8 ± 7.1% vs. 37.0 ± 9.4%; p = 0.035). However, these variables were not significant in the control group (Tables 3 and 4).

The total number of carbohydrate intake (grams/day) was higher at the end of Ramadan in both study arms in comparison to pre-Ramadan which was attributed to breaking the daily fast; the mean total amount of carbohydrate given was 156.2 ± 28.7 g/day versus 211.6 ± 35.3 g/day (p < 0.001) and 161.2 ± 25.3 g/day versus 226.5 ± 39.2 g/day (p < 0.001) in the interventions and control groups, respectively (Table 3). Of note, BMI was comparable between the two groups at study end (p = 0.527).

### Effect of adjunctive vildagliptin therapy on glucometrics and insulin doses delivered by AHCL system during Ramadan

**Fig. 2:**
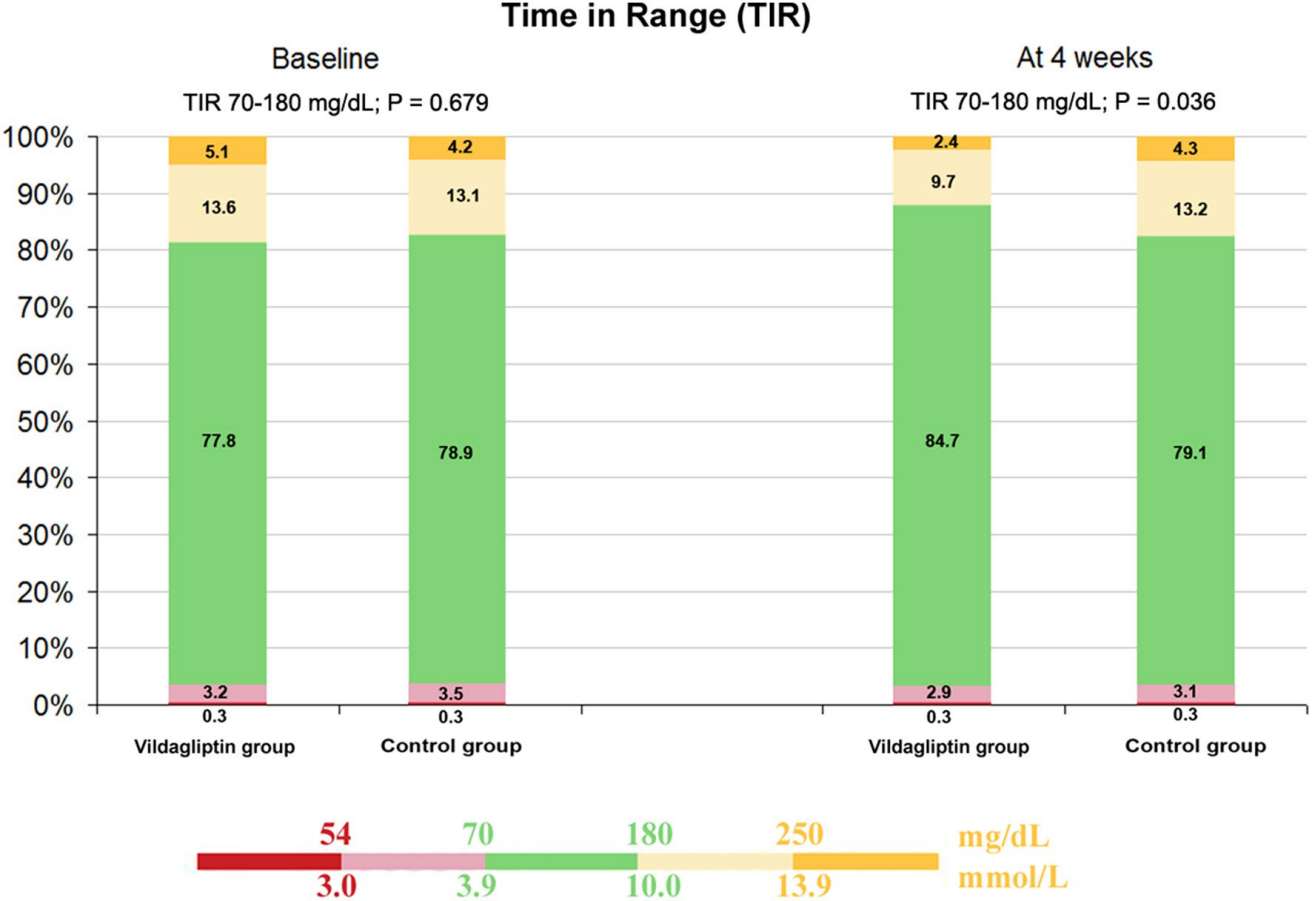
AHCL system performance showing glucose control between vildagliptin and control groups at baseline and at the end of Ramadan among the enrolled patients with type 1 diabetes on MiniMed™ 780G. Glucose values are shown as percentage of time spent in ranges during the study period

As shown in Table 3, the consensus glycemic goals reaching TIR ≥ 70% were obtained during the study period. The intervention cohort demonstrated the highest level in TIR (84.7 ± 8.3% versus 79.1 ± 8.5% for the control arms; p = 0.036) (Fig. 2). Notably, the intervention group did not show any increase in both levels of hypoglycemia before and at end of Ramadan; TBR < 70 mg/dL (< 3.9 mmol/L), 3.2 ± 0.8% versus 2.9 ± 0.9%; p = 0.212 and TBR < 54 mg/dL (< 3.0 mmol/L) 0.3 ± 0.14%% versus 0.3 ± 0.13%; p = 0.875. Correspondingly, hyperglycemia as measured by TAR 180–250 mg/dL (10.0–13.9 mmol/L) and TAR > 250 mg/dL (> 13.9 mmol/L) was also reduced with vildagliptin add-on therapy being 13.6 ± 5.1% versus 9.7 ± 3.6%; p = 0.003 and 5.1 ± 1.3% versus 2.4 ± 0.9; p < 0.001, respectively.

Predictably, no insulin bolus was taken during the daytime because of fasting. However, the ICR during the study phase were made less aggressive in the Iftar meal compared with baseline and was adjusted from 10.2 ± 2.6 g before Ramadan to 11.9 ± 3.4 g at end of Ramadan (p = 0.063) in intervention group while it was made more aggressive and adjusted from 10.8 ± 3.0 g to 7.1 ± 2.9 g (p < 0.001) in control group. Vildagliptin add-on therapy resulted in a significant reduction in the total dose of insulin (on average by 12.8%) being 43.4 ± 8.4 U/day in intervention group and 52.4 ± 9.9 U/day in the control group (p = 0.002) with a mean difference (95% CI) − 6.4 (− 11.714 to − 1.086) U/day compared with 4.1 (− 1.253–9.453) U/day. This reduction of the insulin dose was driven by reducing the number of automated daily correction boluses and decreasing the total bolus dose by 23.9% and 16.3%; p = 0.015 and p < 0.023, respectively (Tables 3 and 4).

Whereas in the control group, there was a significant parallel increase in the total daily dose, total bolus insulin and auto-correction amount per day with high consumption of carbohydrate in non-fasting hours during Ramadan compared with before Ramadan. The adjustment of Iftar meal ICR by the end of the fasting month was decreased from 10.8 ± 3.0 to 7.1 ± 2.9 g (p < 0.001) before Ramadan (i.e. increase the meal bolus from 26.7 ± 7.3 to 32.6 ± 8.7 U/day (p < 0.001). This trend of strengthening the ICR further decreased in the 3rd and 4th weeks of Ramadan in control study arm for better glycemic control of two hours post Iftar meal. The increase of Iftar meal bolus was set aggressively in control groups as majority of Iftar meal contained more than 100 g of carbohydrate.

### AHCL system usability

As regards system usability, sensor adherence was found to be high during Ramadan in both study arms where the overall time spent in closed loop (SmartGuard) by users averaged 98.0 ± 5.1% in Auto Mode. Furthermore, the number of AHCL exits per patient per week was not significant in both study groups when compared before and after Ramadan and also compared between groups during Ramadan (1.1 ± 0.4 versus 1.4 ± 0.7; p = 0.094) indicating confidence in the system’s performance. The most common reasons for exits included ‘‘SmartGuard disabled by user’’, “Sensor was not calibrated”, and ‘‘Sensor Expired’’. Infusion set and reservoir changes were changed on a regular basis (every 3.6 ± 0.6 days for set change and every 3.4 ± 1.1 days for reservoir change on average) and the mean blood glucose calibration per day showed no significant difference between both intervention and control groups (Table 3). In addition, the percentage of time spent in Temp Target of 150 mg/dL (8.3 mmol/L) to mitigate hypoglycemia during fasting hours was comparable between vildagliptin arm and the control group (1.7 ± 0.6% versus 2.1 ± 1.0%; p = 0.114).

### Safety analyses

All of the participants tolerated vildagliptin well and none had any hypersensitivity reactions or adverse events. Importantly, none of the participants had severe hypoglycemia or DKA that required hospitalization. No AHCL system failure was experienced during the study period.

## Discussion

While forefront diabetes technology based on AHCL systems has been proven to be effective in managing overnight and fasting blood glucose levels, it has shown limited efficacy in minimizing post-meal excursions and thus, optimizing overall glycemic control [37]. The delay in insulin absorption as well as its prolonged action that results from the subcutaneous route of insulin delivery leading to exaggerated post-meal hyperglycemic excursions is a major obstacle to attaining post-meal glycemic control [38]. Notably, postprandial glycemia is a significant effector of cardiovascular disease, HbA1c, glycemic variability and mortality in people with diabetes [39, 40]. Even in well-controlled patients, the postprandial period may have a larger adverse impact than sustained fasting hyperglycemia [41].

In a previous publication using open loop insulin pump, a dual bolus with 20% increment given 20 min upfront as a split bolus 70/30 over 4 h was used to achieve physiologic PPG profile in traditional Egyptian Ramadan Iftar meal [20]. To date, only three studies have evaluated the clinical efficacy of AHCL systems in Ramadan. One study [42] showed optimum glycemic control with minimal adjustment of the MiniMed™ 780G AHCL system through increasing the meal bolus for Iftar by a mean of 34.4% for high carbohydrate meals in the non-fasting hours, with more aggressive glycemic settings in AIT and glucose targets.

The other study was a case report on MiniMed 670G hybrid closed-loop system suggested to increase the meal bolus by 10–20%, if the meal contained > 100 g (e.g., to increase the bolus by 20% when 110 g of carbohydrates were eaten, 132 g of carbohydrates was entered into the bolus wizard calculator) and to split bolus insulin 40–50% before and 50–60% after the meal, as the “dual wave” and “square” boluses are disabled in AHCL system [43]. More recently, Wannes et al. [44] showed that MiniMed standard HCL (670G) or AHCL (780G) systems of Medtronic use during Ramadan were safe and were associated with a maintained optimum TIR (> 70%) with no significant hypoglycemia in adolescents and older children with T1DM.

We performed the first randomized controlled trial to investigate the effect of vildagliptin, a DPP-4 inhibitor, on glucose excursions of Iftar Ramadan meals in adolescents and young adults with T1DM during MiniMed™ 780G AHCL system use. The daily glycemic profile was assessed by CGM sensor tracing and we found that vildagliptin as an add-on to insulin delivered by AHCL mainly improved postprandial hyperglycemia, although pre-iftar blood glucose levels were similar in both groups. Based on these findings, it can be suggested that the reduction in SG levels were due to improvement in postprandial hyperglycemia. In fact, we also observed an improvement of glucose variability after vildagliptin therapy. Glucose variability has been reported to be one of the risk factors for cardiovascular diseases [45] and cognitive dysfunction [46, 47]. Therefore, the combination therapy of vildagliptin and insulin given through AHCL system is a beneficial option for the treatment of post-meal glycemic excursions. The use of this adjunctive therapy may ease the burden placed on these AHCL systems to mitigate postprandial glycemic excursions and thereby, achieving lower glycemic excursions with a lower risk of hypoglycemia.

In real-world settings, people with T1DM often omit or delay insulin boluses and miscalculate the carbohydrate content of meals [4]. Early closed-loop systems attempted to alleviate the burden of carbohydrate counting by relying solely on glucose sensor readings to cover meal-related insulin needs, while omitting mealtime insulin boluses. Due to delays in insulin absorption compared with meal glucose absorption, this approach resulted in prolonged postprandial hyperglycemia [48]. Consequently, all current closed-loop systems that outperformed conventional pump therapy require users to input either meal carbohydrate content [49] or meal size category [50, 51].

Here, we used a novel approach to using DDP4- inhibitor to delay meal glucose absorption to match the delays in insulin absorption. Vildagliptin resulted in damping of hyperglycemia in the first 3 h after iftar meal, although a high percentage of TIR was still achieved by AHCL at baseline. To our knowledge, our study is the first attempt to test this novel approach with AHCL during iftar meal in Ramadan. Suboptimal glucose control is associated with imprecise carbohydrate counting, with underestimation being reported as more common than overestimation which might lead to compensatory higher ICRs [4].

A recent clinical trial reported that adolescents using the MiniMed 780G system with a preset personalized fixed carbohydrate amounts can reach international targets of glycemic control. Therefore, it may be a valuable alternative to precise carbohydrate counting in users who are challenged by precise carbohydrate counting. However, meal management with precise carbohydrate counting further improves outcomes and carbohydrate estimation skills remain important with the MiniMed 780G system [52].

In our study, vildagliptin meal intake in adolescents and young adults with T1DM had a better effect than that of rapid-acting insulin analog alone on glycemic peak and time in glycemic range during the 3 h following iftar meal, when used with the AHCL system. Conversely, aspart bolus alone even with more aggressive AHCL settings resulted in a higher postprandial blood glucose levels and a percentage TIR less than that with adjunctive therapy. Vildagliptin effectively replaced first-phase insulin response by lowering glucose levels 60,120 and 180 min from the meal compared with aspart bolus alone.

It has been a difficult challenge for most closed loop systems that do not manually announce meals to effectively control glucose excursions [53]. In our previous study, the aggressive AHCL settings in AIT of 2 h, glucose targets of 100 mg/dL and increasing the meal bolus for Iftar by a mean of 34.4% attained > 80% TIR [42]. Thus, the significant lowering of post-iftar glucose excursions in the present study is a notable finding.

Diabetes management during Ramadan fasting is challenging to the physician in terms of minimizing the risk of hypoglycemia [54]. In this study, supplemental vildagliptin dosing during iftar meal, according to postprandial glucose, has been shown to increase TIR in the absence of an increase in hypoglycemic events. However, the contemporary use of AHCL for automated insulin adjustment was expected to overcome supplemental bolusing with the ultimate intent of simplifying meal daily management and increase therapeutic compliance in a real world setting. Of note, we found that neither AHCL alone or with added vildagliptin therapy increased the risk for late hypoglycemia during the 3 h after iftar meal.

Furthermore, in a previous real-life study in Egypt, treatment with vildagliptin was associated with lower incidence of hypoglycemia compared with sulfonylurea and showed good glycemic and weight control in patients with T2DM fasting during Ramadan [55]. Conversely to other oral hypoglycemic agents and sulfonylureas, a research review has collected evidence-based clinical trials and observations that DPP-4 inhibitors such as vildagliptin minimized the risk of hypoglycemia during Ramadan fasting and have also shown higher treatment adherence with better patient compliance and glycemic control. Of notice, this drug did not require any treatment modifications during Ramadan [54, 55].

The important finding of this study is that add-on 50 mg/day vildagliptin in patients on AHCL therapy, resulted in a significant improvement of glycemic control where GMI decreased by about 7.5% from the baseline. This improvement is greater than previous studies that investigated the effect of the combination of vildagliptin and insulin injection in Western countries [56, 57] and in Japan [58]. However, a greater fall in GMI could be observed in patients with higher baseline GMI levels than our cohort. For this reason, the reduction in GMI recorded in our study could be considered high and reasonable because of the low baseline GMI levels.

The current study provides evidence that the positive effect of an adjunct therapy to insulin therapy with the DDP-4 inhibitor is maintained even if the most advanced therapy for T1DM is used. Vildagliptin exhibited a pronounced increase in TIR by 8.8% (127 min/day) despite less aggressive ICR settings in the intervention group, both in adolescents and young adults. In line with the glucose-lowering effect of this class of drugs, insulin requirements was reduced in the intervention group, with this effect being driven by a reduction in both bolus insulin and automated bolus corrections. Thus, this study provides evidence that young patients with T1DM will probably benefit from adjunct therapy with vildagliptin combined with automated insulin delivery, as current AHCL systems often fail to reach target TIR during Ramadan, in particular postprandial TIR targets. However, during carbohydrate, fat and protein-rich meals used in this real world study, manual pre-prandial bolus administration will still be necessary, even with combined therapy of AHCL and DDP4- inhibition.

Furthermore, an increasingly large proportion of pediatric and adult patients with T1DM are overweight or obese, which in turn contributes to problems in achieving optimal metabolic control and increases the risk of future cardiovascular disease [59]. The recent failure of metformin to improve metabolic control of obese adolescents with T1DM diabetes [60] illustrates a continuing unmet need for an adjunctive therapy like DDP-4 inhibitors that could reduce insulin requirements in type 1 diabetes. In addition, our data also suggest that the major benefit of these agents as adjunctive treatment may be for concomitant increases in insulin sensitivity. The lower system set point (target 100 mg/dL) used in the study may also explain adjusting ICR to be less aggressive within the intervention group to minimize frequency of hypoglycemia.

Reaching glycemic targets for glucose control is challenging especially in adolescents and young adults and in a complex meal like Iftar. This study provides evidence of an additional positive effect of adjunctive therapy of a similar dimension to that observed in the regulatory trials in adults on multiple daily injections (MDI) and continuous subcutaneous insulin infusion (CSII). Notably, during none fasting hours, despite complexity of iftar meals, a highly significant improvement of TIR was achieved with vildagliptin compared with the control group, which is well above the 5% change that was considered to be clinically significant in the recent consensus statement [61]. The results regarding the efficacy of glucose control compare favorably with those from studies performed with the same system without adjunct therapy [62, 63].

In the study, the add-on vildagliptin therapy showed a comparable time frame reaching the target range, apparently with less variability, a lower mean TAR and near normal postprandial values after ‘iftar bolus’ in real life. Despite the near-normoglycemic glucose control achieved with AHCL, a further improvement of TIR was possible, which was on average even higher than that observed in other clinical trials involving adjunctive therapies such as SGLT inhibitors [64–66]. In addition to lowering glucose variability, especially during the non-fasting, adding vildagliptin to complement AHCL insulin therapy could represent another step towards fully closing the loop.

We found that vildagliptin add-on therapy was safe and well tolerated in our patients with type 1 diabetes. The safety of vildagliptin has been reported in several studies [54, 58, 67]. The reported safety of this drug is unlike SGLT inhibitors which although improves glycemic control but increases ketone concentration and DKA [68, 69]. Sodium-glucose-linked cotransporter inhibitors have been extensively researched in T1DM, with average reductions in placebo-adjusted HbA1c by 0.39%, and total daily dose of insulin by approximately 10%. Unfortunately, many trials revealed an increased risk of DKA, as high as 5 times the relative risk compared to placebo. Benefit to risk ratio in these possible adjunct oral drugs to closed-loop insulin therapy should be improved [68, 69].

Previous studies reported AHCL systems often fail to reach target TIR during daytime, in particular postprandial TIR targets [13, 70]. The DEPICT trials showing that with dapagliflozin, the rate of DKA was higher in patients on insulin pump than on multiple daily injections. In particular, classical insulin pump related issues caused most of the DKA cases, and should be the focus of education if used with full closed loop [64].

The main strength of this study is the novelty of combining an AHCL system with a DDP-4 inhibitor for an actual iftar meal in place of mixed-meal which makes our observations generalizable in a real world setting. Limitations of this study include the relatively small sample size; however, this was a single-center study. Nevertheless, larger studies are required to validate our results and determine whether adjunctive therapy requirements remain constant across solid ‘real-world’ Ramadan iftar meals.

In conclusion, our study showed that 50 mg/day vildagliptin as an add-on therapy to MiniMed™ 780G AHCL system in Ramadan iftar meal improved both glycemic control and glucose fluctuation in Egyptian patients with T1DM without increase in hypoglycemia. AHCL treatment with iftar meal vildagliptin was safe and well tolerated and provided superior postprandial glucose control compared with announced pre-meal aspart boluses mitigating postprandial hyperglycemia to account for the glucodynamic action profile. The combination of AHCL with adjunct DDP-4 inhibitor therapy, as shown in this trial, may constitute a novel approach to maximize TIR during closed loop therapy. Our results extend the evidence that, regardless of the insulin delivery method, adjunctive DDP-4 therapy has great potential to help individuals with T1DM achieve meaningful clinical benefits beyond improvement in TIR. Multicenter studies of AHCL system with these adjunctive agents are needed to determine their full efficacy and safety profiles in special settings such as Ramadan.

## Data Availability

The data that support the findings of this study are available from the corresponding author upon reasonable request.
